# Supplementary material for: Expectations of reward and efficacy guide cognitive control allocation
Source: Nat Commun. 2021 Feb 15;12:1030. doi: 10.1038/s41467-021-21315-z (PMC7884731; doi:10.1038/s41467-021-21315-z)
Supplement: Supplementary file 1 — Supplementary Information [file 41467_2021_21315_MOESM1_ESM.pdf]

Supplementary Information

Expectations of reward and efficacy guide cognitive control allocation

Frömer, R. *, Lin, H.*, Dean Wolf, C. K., Inzlicht, M., & Shenhav, A.

Supplementary Table 1. *Effects of Reward and Efficacy on Performance – Study 1*

	**Accuracy**			**Accurate RT**		
Predictors	*Log-Odds*	*CI*	*p*	*Estimates*	*CI*	*p*
(Intercept)	2.16	1.87 – 2.45	**<0.001**	600.30	581.46 – 619.13	**<0.001**
Efficacy	0.10	-0.06 – 0.26	0.214	-14.55	-20.63 – -8.46	**<0.001**
Reward	0.05	-0.11 – 0.21	0.543	-9.81	-15.89 – -3.73	**0.002**
Congruency n-i	0.44	0.17 – 0.71	**0.001**	-55.99	-68.83 – -43.16	**<0.001**
Congruency c-n	0.54	0.29 – 0.78	**<0.001**	-15.80	-23.63 – -7.97	**<0.001**
Trial	0.13	0.05 – 0.21	**0.002**	-1.53	-4.58 – 1.53	0.327
Efficacy: Reward	0.15	-0.17 – 0.46	0.357	-9.75	-21.92 – 2.41	0.116
Observations	6182			5435		

Note: Statistics are derived from linear mixed effects models with predictors as noted. Statistically significant p-values (< 0.05, two-sided) are displayed in bold. Congruency (n-i) refers to the comparison between incongruent and neutral Stroop stimulus; Congruency (c-n) refers to the comparison between neutral and congruent Stroop stimulus.

Supplementary Table 2. *Effects of Reward and Efficacy on Performance – Study 2*

	**Accuracy**			**Accurate RT**		
Predictors	*Log-Odds*	*CI*	*p*	*Estimates*	*CI*	*p*
(Intercept)	1.86	1.65 – 2.08	**<0.001**	648.43	631.27 – 665.58	**<0.001**
Efficacy	0.08	0.01 – 0.15	**0.033**	-5.89	-10.70 – -1.08	**0.016**
Reward	0.01	-0.07 – 0.08	0.885	-5.03	-8.50 – -1.56	**0.004**
Congruency n-i	0.65	0.44 – 0.85	**<0.001**	-66.17	-76.64 – -55.69	**<0.001**
Congruency c-n	0.34	0.18 – 0.50	**<0.001**	-15.23	-20.63 – -9.83	**<0.001**
Trial	0.04	0.00 – 0.07	**0.049**	-10.87	-12.61 – -9.12	**<0.001**
Efficacy: Reward	0.02	-0.13 – 0.16	0.791	-9.23	-16.16 – -2.29	**0.009**
Observations	24404			20510		

Note: Statistics are derived from linear mixed effects models with predictors as noted. Statistically significant p-values (< 0.05, two-sided) are displayed in bold. Congruency (n-i) refers to the comparison between incongruent and neutral Stroop stimulus; Congruency (c-n) refers to the comparison between neutral and congruent Stroop stimulus.

Supplementary Note 1.

In addition to the within-study effects reported above, we also saw an overall difference in performance between the studies. Relative to Study 1, participants in Study 2 were slower overall ($b = -51.95$, $p <.001$) and less accurate ($b = -0.50$, $p = .013$), which could be attributable to differences in task design or participant pools.

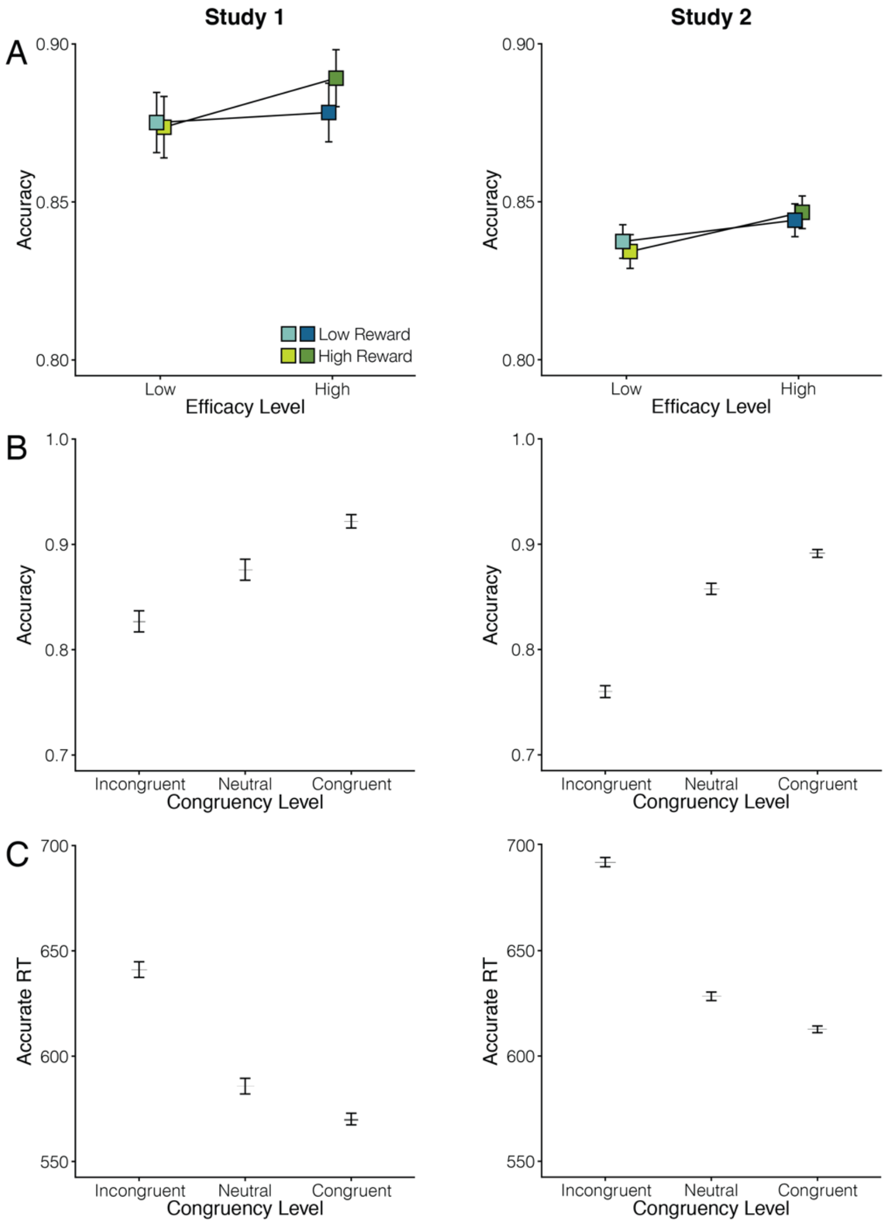

Supplementary Figure 1. Performance effects across both Studies. Left: Study 1, n = 21 participants, Right: Study 2, n = 44 participants. **A.** Effects of Reward and Efficacy on performance accuracy. **B.** Effect of Stroop congruency on accuracy. **C.** Effects of Stroop Congruency on accurate RT. **A. – C.** Error bars represent within-subject standard error of the mean.

Supplementary Table 3. *Between-Study Comparison of Behavioral Effects*

	**Accuracy**			**Accurate RT**		
Predictors	*Log-Odds*	*CI*	*p*	*Estimates*	*CI*	*p*
(Intercept)	2.11	1.91 – 2.31	**<0.001**	624.25	609.99 – 638.51	**<0.001**
Efficacy	0.09	0.00 – 0.17	**0.047**	-10.26	-15.01 – -5.51	**<0.001**
Reward	0.03	-0.06 – 0.12	0.503	-7.52	-11.23 – -3.81	**<0.001**
Congruency n-i	0.53	0.35 – 0.72	**<0.001**	-61.12	-70.24 – -52.00	**<0.001**
Congruency c-n	0.45	0.29 – 0.60	**<0.001**	-15.54	-20.87 – -10.22	**<0.001**
Trial	0.08	0.04 – 0.13	**<0.001**	-4.01	-5.99 – -2.03	**<0.001**
Efficacy: Reward	0.08	-0.09 – 0.25	0.370	-9.44	-16.87 – -2.01	**0.013**
Study	-0.50	-0.89 – -0.11	**0.013**	51.95	23.43 – 80.47	**<0.001**
Efficacy: Study	-0.02	-0.19 – 0.15	0.816	8.61	-0.88 – 18.10	0.075
Reward: Study	-0.05	-0.23 – 0.12	0.560	4.97	-2.46 – 12.40	0.190
Congruency n-i : Study	0.24	-0.12 – 0.61	0.193	-10.11	-28.35 – 8.13	0.277
Congruency c-n : Study	-0.23	-0.53 – 0.07	0.140	0.66	-10.00 – 11.31	0.904
Trial : Study	-0.13	-0.22 – -0.03	**0.009**	-4.53	-8.48 – -0.57	**0.025**
Efficacy: Reward:Study	-0.12	-0.47 – 0.22	0.482	0.42	-14.45 – 15.28	0.956
N	65 SubID			65 SubID		
Observations	30566			25945		

Note: Statistics are derived from linear mixed effects models with predictors as noted. Statistically significant p-values (< 0.05, two-sided) are displayed in bold. Congruency (n-i) refers to the comparison between incongruent and neutral Stroop stimulus; Congruency (c-n) refers to the comparison between neutral and congruent Stroop stimulus.

Supplementary Table 4. *Parametric Effects of Reward and Efficacy on Performance – Study 3*

	**Accuracy**			**Accurate RT**		
Predictors	*Log-Odds*	*CI*	*p*	*Estimates*	*CI*	*p*
(Intercept)	1.78	1.51 – 2.04	**<0.001**	602.99	588.27 – 617.71	**<0.001**
Efficacy	-0.01	-0.06 – 0.04	0.746	-3.85	-6.10 – -1.60	**0.001**
Reward	0.03	-0.01 – 0.08	0.167	-7.02	-9.24 – -4.81	**<0.001**
Congruency n-i	0.28	0.13 – 0.42	**<0.001**	-52.34	-66.37 – -38.31	**<0.001**
Congruency c-n	0.55	0.37 – 0.73	**<0.001**	-18.15	-24.81 – -11.48	**<0.001**
Trial	0.08	0.03 – 0.14	**0.003**	-4.39	-6.88 – -1.90	**0.001**
Efficacy:Reward	0.01	-0.03 – 0.05	0.621	-2.27	-4.27 – -0.26	**0.027**
N	35			35		
Observations	10251			8531		

Note: Statistics are derived from linear mixed effects models with predictors as noted. Statistically significant p-values (< 0.05, two-sided) are displayed in bold. Congruency (n-i) refers to the comparison between incongruent and neutral Stroop stimulus; Congruency (c-n) refers to the comparison between neutral and congruent Stroop stimulus.

Supplementary Table 5. *ERPs predict behavior within incentive conditions*

	Accuracy			Accurate RT		
Predictors	*Log-Odds*	*CI*	*p*	*Estimates*	*CI*	*p*
(Intercept)	1.87	1.65 – 2.08	**<0.001**	647.64	630.60 – 664.68	**<0.001**
Efficacy	0.08	0.01 – 0.16	**0.034**	-3.79	-8.62 – 1.04	0.124
Reward	0.03	-0.05 – 0.10	0.455	-2.99	-6.59 – 0.61	0.104
Congruency n-i	0.65	0.44 – 0.85	**<0.001**	-63.99	-68.85 – -59.13	**<0.001**
Congruency c-n	0.34	0.18 – 0.49	**<0.001**	-15.82	-20.23 – -11.41	**<0.001**
Baseline	0.02	-0.02 – 0.07	0.333	2.24	0.05 – 4.43	**0.045**
Trial	0.04	0.00 – 0.08	**0.039**	-10.79	-12.60 – -8.98	**<0.001**
Efficacy: Reward	0.01	-0.14 – 0.16	0.905	-9.85	-17.04 – -2.66	**0.007**
$Efficacy_l$: $Reward_l$: P3b	0.05	-0.03 – 0.12	0.223	-11.33	-15.05 – -7.62	**<0.001**
$Efficacy_h$: $Reward_l$: P3b	0.16	0.08 – 0.24	**<0.001**	-4.89	-8.50 – -1.28	**0.008**
$Efficacy_l$: $Reward_h$: P3b	-0.01	-0.09 – 0.07	0.801	-4.53	-8.23 – -0.84	**0.016**
$Efficacy_h$: $Reward_h$: P3b	0.10	0.03 – 0.18	**0.009**	-7.52	-11.11 – -3.94	**<0.001**
$Efficacy_l$: $Reward_l$: CNV	-0.06	-0.14 – 0.02	0.117	16.50	11.72 – 21.28	**<0.001**
$Efficacy_h$: $Reward_l$: CNV	-0.08	-0.16 – -0.00	**0.045**	13.28	8.50 – 18.05	**<0.001**
$Efficacy_l$: $Reward_h$: CNV	-0.10	-0.18 – -0.03	**0.007**	13.89	9.10 – 18.68	**<0.001**
$Efficacy_h$: $Reward_h$: CNV	-0.15	-0.23 – -0.07	**<0.001**	18.68	13.96 – 23.41	**<0.001**
N	44			44		
Observations	22580			18999		

Note: Statistics are derived from linear mixed effects models with predictors as noted. Statistically significant p-values (< 0.05, two-sided) are displayed in bold.

Supplementary Table 6. *Incentive effects on pupil dilation*

	Pupil Response		
Predictors	*Estimates*	*CI*	*p*
(Intercept)	0.28	0.22 – 0.35	**<0.001**
Efficacy	-0.06	-0.08 – -0.04	**<0.001**
Reward	0.01	-0.01 – 0.03	0.243
Trial	-0.19	-0.28 – -0.10	**<0.001**
Efficacy * Reward	-0.02	-0.05 – 0.02	0.309
Observations	19986		

Note: Statistics are derived from linear mixed effects models with predictors as noted. Statistically significant p-values (< 0.05, two-sided) are displayed in bold.

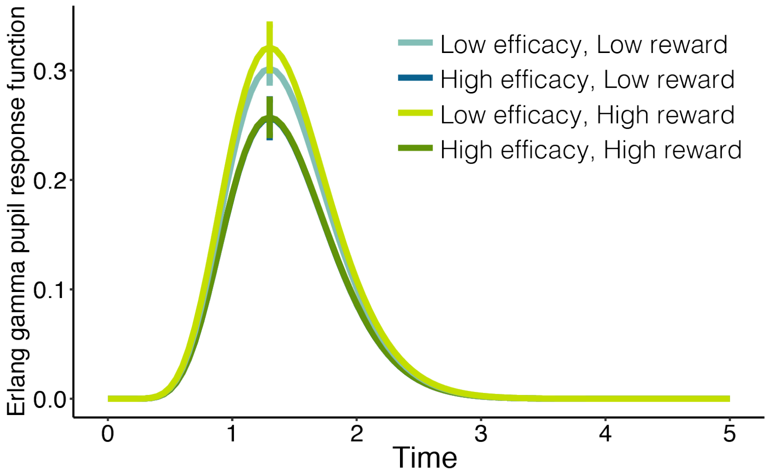

Supplementary Figure 2. Pupillary response increases with uncertainty, not motivation. Cue-related pupillary response estimated using deconvolution as a function of incentive condition. Error bars represent the 95% CI of the scaling parameters. n = 44 participants.

Supplementary Note 2. Response-related signals reflect violations of performance criteria and expectations

We tested how incentives would modulate response and outcome evaluation. To index response evaluation, we analyzed the error related negativity (ERN). This signal is sensitive to response errors and has been proposed to reflect reward prediction errors computed based on the change in expected outcome as a function of internal evaluations of the response (Holroyd & Coles, 2002). In our task, performance was rewarded if participants were both fast and accurate. Hence, internal response evaluations should be sensitive to both the accuracy and speed of the submitted response, with larger ERN amplitudes for both incorrect and slower responses. Consistent with this assumption, we found additive effects of response accuracy and RT (Supplementary Table 5). ERN amplitude was larger for errors compared to correct responses, and increased with increasing response time. In addition to the linear increase of ERN amplitude with RT, we also observed a quadratic component, such that RT effects tapered off around the time of the criterion (~750 ms, cf. Fig. 3). In addition, we tested whether participants were sensitive to incentive-dependent expectations about their response accuracy. To do so, we computed the average accuracy within each incentive condition (probability correct for each combination of efficacy and reward). We found that higher incentive-dependent accuracy expectations, amplified accuracy effects on the ERN (Fig. 3). Congruency effects on ERN amplitude were modulated by RT, such that differences in ERN as a function of congruency conditions washed out for longer RTs (Fig. 3).

As reported in the main manuscript, when testing our prediction that higher reward and efficacy would amplify internal monitoring, we found the expected 3-way interaction between efficacy, reward, and accuracy (Supplementary Table 5). However, at odds with our prediction, the effect was driven by largest ERN amplitude on error trials in the condition in which both efficacy and reward were low (Supplementary Table 6), and this pattern was suspiciously mirrored in error RTs (Supplementary Table 7 and Fig. 3).

While this pattern of activity was not predicted (we predicted largest ERN amplitudes to errors on high reward, high efficacy trials), it is notable that it mirrors the pattern we observed for error

RTs (Fig. 3), where low-reward/low-efficacy trials were associated with the fastest errors (consistent with effort minimization by, e.g., responding with any key; b = 29.08, p = .001; Supplementary Table 7). Speculatively, these findings may reflect a shift from more effortful proactive to less effortful reactive control as the expected value of proactive control decreased (Braver, 2012). Thus, participants may have responded more impulsively, and then evaluated those responses immediately after they were executed. On the large proportion of incongruent error trials participants may further have re-evaluated the value of control in a reactive manner given the new information about the difficulty of the trial. Future research will need to test specific predictions of this ad-hoc interpretation in a principled manner. Given the sensitivity of the ERN to multiple aspects of performance and performance expectations, it is possible that the complex interaction pattern reflects different mixtures of these multiple factors across incentive conditions.

Supplementary Table 7. *Reward, Efficacy, and Performance Effects on ERN*

	**ERN**		
Predictors	*Estimates*	*CI*	*p*
(Intercept)	2.95	1.19 – 4.71	**0.001**
Efficacy	0.09	-0.14 – 0.33	0.444
Reward	0.03	-0.20 – 0.26	0.773
Accuracy	4.31	3.56 – 5.07	**<0.001**
Congruency n-i	7.87	4.00 – 11.73	**<0.001**
Congruency c-n	-4.76	-8.35 – -1.17	**0.009**
RT	-7.59	-12.48 – -2.71	**0.002**
RT quadratic	4.57	0.81 – 8.34	**0.017**
Mean Accuracy (mAcc)	-1.96	-5.02 – 1.11	0.210
Baseline	-0.36	-0.38 – -0.35	**<0.001**
Efficacy: Reward	-0.62	-1.08 – -0.15	**0.009**
Efficacy: Accuracy	-0.07	-0.54 – 0.40	0.755
Reward: Accuracy	-0.36	-0.82 – 0.10	0.127
Congruency n-i : RT	-19.62	-31.37 – -7.88	**0.001**
Congruency c-n : RT	11.54	0.38 – 22.70	**0.043**
Congruency n-i : RT qu.	11.50	2.80 – 20.20	**0.010**
Congruency c-n : RT qu.	-6.28	-14.73 – 2.16	0.145
Accuracy : mAcc	7.67	2.24 – 13.11	**0.006**
Efficacy: Reward: Accuracy	1.52	0.60 – 2.45	**0.001**
N_{SubID}	44		
Observations	22954		

Note: Statistics are derived from linear mixed effects models with predictors as noted. Statistically significant p-values (< 0.05, two-sided) are displayed in bold. Congruency (i-n) refers to the comparison between incongruent and neutral Stroop stimulus; Congruency (n-c) refers to the comparison between neutral and congruent Stroop stimulus.

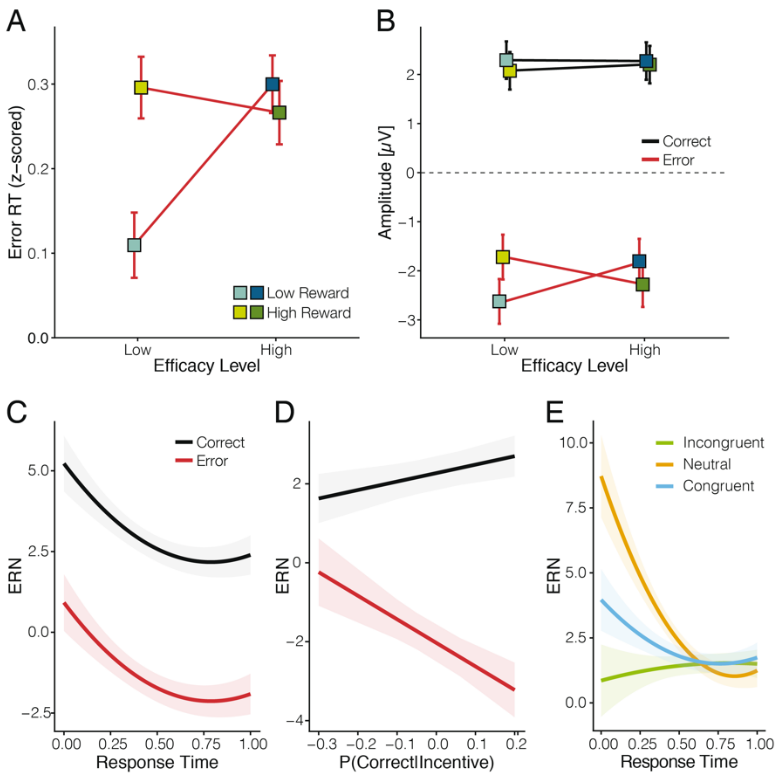

Supplementary Figure 3. Response-evaluation is reflects violations of performance expectations, not incentive-driven outcome salience. A. Error RTs are plotted as a function of incentive conditions and show the same pattern as ERN amplitudes. **B.** Response-locked amplitudes are plotted as a function of response accuracy and incentive conditions. ERN amplitude is largest on error trials with low reward and low efficacy. **C.** Fixed effects of accuracy and response time on response- locked activity. Response-locked amplitudes are more negative for errors, as well as slower responses. **D.** Fixed effects of accuracy in interaction with incentive-wise mean accuracy. Amplitudes for correct and incorrect responses are more similar when errors are more frequent. Error-related activity is more negative when errors are less frequent. **E**. Fixed effects of Congruency in interaction with RT. Congruency effects on the ERN wash out for longer response times. **B.-E.** Predicted effects from the linear mixed effects model. Shaded and regular error bars represent standard error of the mean. n = 44 participants.

Supplementary Table 8. *Incentive and Performance Effects on ERN Amplitude in Correct and Error Trials*

	ERN error			ERN correct		
Predictors	*Estimates*	*CI*	*p*	*Estimates*	*CI*	*p*
(Intercept)	2.39	-1.66 – 6.43	0.248	4.48	2.54 – 6.42	**<0.001**
Efficacy	0.12	-0.32 – 0.57	0.582	0.05	-0.13 – 0.24	0.581
Reward	0.19	-0.24 – 0.63	0.382	-0.14	-0.33 – 0.04	0.120
Congruency2-1	-2.38	-11.63 – 6.88	0.615	10.56	6.29 – 14.83	**<0.001**
Congruency3-2	-2.09	-11.21 – 7.03	0.653	-5.50	-9.42 – -1.59	**0.006**
RT	-13.53	-25.21 – -1.85	**0.023**	-5.54	-10.92 – -0.16	**0.044**
RT quadratic	9.66	1.08 – 18.24	**0.027**	2.93	-1.22 – 7.08	0.167
meanCorr1	-5.42	-10.74 – -0.09	**0.046**	1.57	-1.46 – 4.61	0.310
Baseline	-0.37	-0.40 – -0.34	**<0.001**	-0.36	-0.37 – -0.35	**<0.001**
Efficacy: Reward	-1.34	-2.21 – -0.47	**0.003**	0.15	-0.22 – 0.51	0.432
Congruency2-1 * RT	8.12	-19.67 – 35.92	0.567	-27.30	-40.31 – -14.29	**<0.001**
Congruency3-2 * RT	3.95	-23.93 – 31.82	0.781	13.81	1.60 – 26.02	**0.027**
Congruency2-1 * RT qu.	-7.08	-27.44 – 13.27	0.495	16.87	7.21 – 26.54	**0.001**
Congruency3-2 * RT qu.	-0.41	-21.16 – 20.34	0.969	-8.07	-17.34 – 1.20	0.088
N	44 SubID			44 SubID		
Observations	3640			19314		

Note: Statistics are derived from linear mixed effects models with predictors as noted. Statistically significant p-values (< 0.05, two-sided) are displayed in bold. Congruency (i-n) refers to the comparison between incongruent and neutral Stroop stimulus; Congruency (n-c) refers to the comparison between neutral and congruent Stroop stimulus.

Supplementary Table 9. *Incentive Effects on Error RT*

	Error RT		
Predictors	*Estimates*	*CI*	*p*
(Intercept)	682.12	660.66 – 703.57	**<0.001**
Reward	9.45	1.20 – 17.70	**0.025**
Congruency n-i	-47.70	-60.83 – -34.57	**<0.001**
Congruency c-n	10.16	-8.41 – 28.74	0.284
Trial	-10.11	-14.22 – -6.00	**<0.001**
Reward [l] : Efficacy	29.08	14.24 – 43.92	**<0.001**
Reward[h] : Efficacy	-4.51	-19.33 – 10.31	0.551
N SubID	44		
Observations	3875		

Note: Statistics are derived from linear mixed effects models with predictors as noted. Statistically significant p-values (< 0.05, two-sided) are displayed in bold. Congruency (i-n) refers to the comparison between incongruent and neutral Stroop stimulus; Congruency (n-c) refers to the comparison between neutral and congruent Stroop stimulus.

Supplementary Note 3.
In addition to ERN amplitude, we analyzed response-locked theta power, which indexes similar, although not functionally identical processes to the ERN (Beatty, Buzzell, Roberts, & McDonald, 2020). We had predicted that midfrontal theta following the response would be largest when participants make errors on high reward and efficacy trials. Our results (Supplementary Table 8) are partially in line with our predictions, such that theta power was higher when participants made errors on high efficacy trials, reflected in a significant interaction of efficacy and accuracy (b = -276.12, p = .013), as well reliably higher theta power for high compared to low efficacy on error, but not correct trials (error: b = 318.45, p = .002, correct: b = 42.33, p = .337). However, we observed no reliable effects of reward or interactions with reward and if anything theta power was lower on high relative to low reward trials.

Supplementary Table 10. *Incentive Effects on Error Processing in Midfrontal Theta*

	**Theta Power**		
Predictors	*Estimates*	*CI*	*p*
(Intercept)	4337.09	3912.50 – 4761.68	**<0.001**
Efficacy	180.39	71.12 – 289.66	**0.001**
Reward	-54.11	-163.00 – 54.77	0.330
Accuracy	-1529.32	-1935.98 – -1122.66	**<0.001**
Baseline	212.27	169.27 – 255.28	**<0.001**
Efficacy: Accuracy	-276.12	-494.68 – -57.57	**0.013**
Reward: Accuracy	166.43	-51.34 – 384.20	0.134
Observations	23251		

Note: Statistics are derived from linear mixed effects models with predictors as noted. Statistically significant p-values (< 0.05, two-sided) are displayed in bold.

Supplementary Note 4. Feedback evaluation reflects outcome magnitude and predictability
We had predicted that incentives would shape feedback processing, such that reward effects would be amplified under high efficacy. To test this prediction, we quantified the magnitude of the FRN peak-to-peak relative to the preceding positive deflection. This measure eliminates baseline confounds that may lead to spurious effects (e.g. due to anticipatory signals). Consistent with the reward prediction error account of the FRN (Holroyd & Coles, 2002), the peak-to-peak FRN reflected reward receipt versus omission, with larger FRN amplitudes for unrewarded compared to rewarded trials (b = 0.80, p <.001), as well as stronger effects for larger rewards (b = 0.81, p = .007). However, in addition, this measure revealed an interaction of reward receipt with Efficacy, such that reward effects were reduced for high compared to low efficacy (b = -0.83, p = .007). While at odds with our original prediction, this finding is plausible, given that under high efficacy rewards were controllable, and could be predicted based on performance (cf. response evaluation), thus, the impact of these rewards was reduced.

Supplementary Table 11. *Incentive Modulations of Reward Processing (FRN)*

	Peak to peak FRN amplitude		
Predictors	*Estimates*	*CI*	*p*
(Intercept)	-34.97	-37.29 – -32.64	**<0.001**
Is Rewarded y-n	0.80	0.41 – 1.19	**<0.001**
Efficacy	-0.00	-0.30 – 0.30	0.996
Reward	0.07	-0.23 – 0.37	0.655
Congruency n-i	-0.08	-0.46 – 0.30	0.691
Congruencyc-n	0.09	-0.27 – 0.44	0.631
Trial	-0.52	-0.66 – -0.38	**<0.001**
Is Rewarded y-n:Efficacy	-0.83	-1.43 – -0.23	**0.007**
Is Rewarded y-n:Reward	0.81	0.22 – 1.41	**0.007**
Observations	22721		

Note: Statistics are derived from linear mixed effects models with predictors as noted. Statistically significant p-values (< 0.05, two-sided) are displayed in bold. Congruency (n-i) refers to the comparison between incongruent and neutral Stroop stimulus; Congruency (c-n) refers to the comparison between neutral and congruent Stroop stimulus.

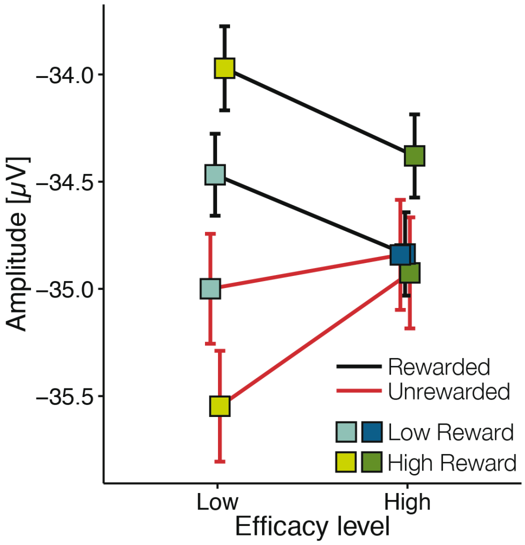

Supplementary Figure 4. Outcome effects on FRN amplitude are larger for larger rewards, but lower efficacy. Mean peak-to-peak FRN amplitude as a function of reward receipt vs. omission, reward magnitude, and efficacy on FRN amplitude. Error bars represent standard errors of the mean. n = 44 participants.

Supplementary Discussion

Our ERN results - largest amplitudes on error trials with low reward and low efficacy - are inconsistent with a motivational salience account. Instead we found response-locked activity that tracked violations of either performance criterion (accuracy or response speed): an ERN to errors compared to correct responses, as well as more negative amplitudes to longer RTs, maximal around the time of the reward deadline (Fig. 3, Supplementary Table 4). ERN amplitudes were also larger when errors were less expected based on one's average performance in a given incentive condition (i.e., when performing well overall) (Brown & Braver, 2005; Hughes & Yeung, 2011).

Our results at the time of feedback (FRN) - reduced effects of reward receipt vs omission under high efficacy - are also inconsistent with a motivational salience account. Instead, they indicate that predictability modulated outcome evaluation. Under high (but not low) efficacy, outcome information is redundant to the degree that participants can internally evaluate their performance, as suggested by our ERN results and previous work (Bellebaum & Colosio, 2014; Bultena, Danielmeier, Bekkering, & Lemhöfer, 2017; Frömer, Nassar, Stürmer, Sommer, & Yeung, 2018; Holroyd & Coles, 2002), reducing the response to feedback. As difficulty manipulations are known to influence such internal evaluations (Boldt, de Gardelle, & Yeung, 2017), our results call for caution when interpreting corresponding increases (Hernandez Lallement et al., 2014; Ma, Meng, Wang, & Shen, 2014; Wang, Zheng, & Meng, 2017), or decreases (effort discounting; Botvinick, Huffstetler, & McGuire, 2009) in outcome valuation as effort-related. Taken together, the observed sensitivity of ERN and FRN to surprising evaluative information is in line with the reward prediction error account of the ERN and FRN(Holroyd & Coles, 2002), and emphasizes the interaction of internal performance monitoring and feedback processing(Frömer et al., 2018). Thus, in contrast to the robust incentive-driven increase in markers of proactive control, our results are inconsistent with incentive-driven increases in motivational salience of outcomes, reactive control, or overall engagement.

With that said, there was little use for incentive-based reactive control in the present paradigm, as feedback was only informative about the success on the current, but not the value of effort on the subsequent trial. This difference in functional utility of feedback may explain why our results square with previously reported larger FRN amplitudes for more reliable feedback. When participants were led to believe that some equally unreliable feedback could be used to improve task performance – i.e. that reactive control would be more efficacious – their FRN amplitudes to that feedback were larger (Muhlberger, Angus, Jonas, Harmon-Jones, & Harmon-Jones, 2017; Schiffer, Siletti, Waszak, & Yeung, 2017). Thus, effort cost-benefit analyses, determining the intensity *and* type of the optimal control signal, may yield different results for proactive and reactive control signals, depending on the task (Braver, 2012; Shenhav, Botvinick, & Cohen, 2013; Shenhav, Cohen, & Botvinick, 2016). Thus, additional work is needed to investigate under which conditions and how reward and efficacy shape reactive trial-to-trial adjustments of control.

Supplementary Table 12. *Performance and Reward Summary Statistics*

	Fast and accurate	Rewarded
**Study 1**	M = 0.77, SD = 0.10	M = 0.76, SD = 0.10
**Study 2**	M = 0.61, SD = 0.16	M = 0.60; SD = 0.15

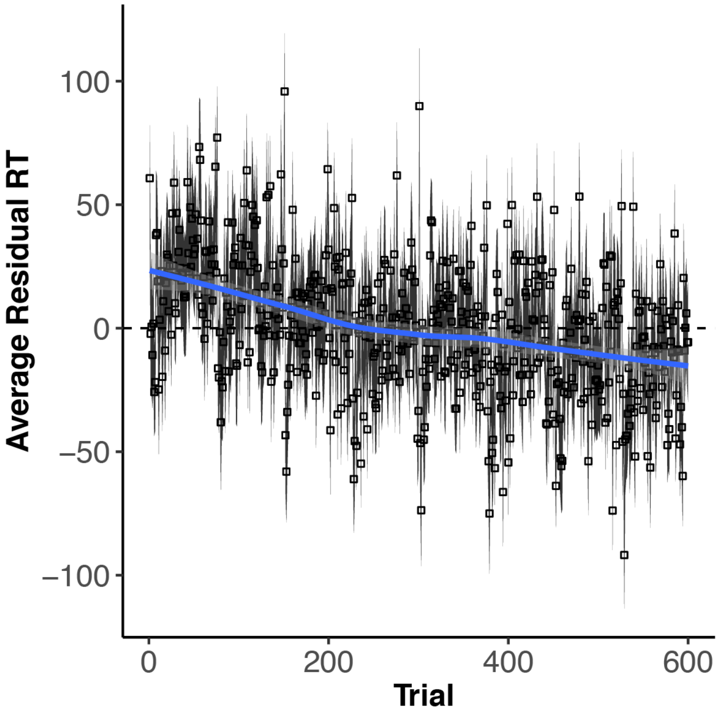

Supplementary Figure 5. Residuals from model without trial regressors reveal approximately linear changes over the course of the experiment. Plotted are the average residuals for each trial in Study 2 with standard errors as shaded error bars. Superimposed is an unconstrained spline fit (blue line) that yields an approximately linear trend. n = 44 participants.

References

Beatty, P. J., Buzzell, G. A., Roberts, D. M., & McDonald, C. G. (2020). Contrasting time and frequency domains: ERN and induced theta oscillations differentially predict post-error behavior. *Cognitive, Affective, & Behavioral Neuroscience*. doi:10.3758/s13415-020-00792-7

Bellebaum, C., & Colosio, M. (2014). From Feedback- to Response-based Performance Monitoring in Active and Observational Learning. *J Cogn Neurosci, 26*(9), 2111-2127. doi:10.1162/jocn_a_00612 %M 24666168

Boldt, A., de Gardelle, V., & Yeung, N. (2017). The Impact of Evidence Reliability on Sensitivity and Bias in Decision Confidence. *J Exp Psychol Hum Percept Perform*. doi:10.1037/xhp0000404

Botvinick, M. M., Huffstetler, S., & McGuire, J. T. (2009). Effort discounting in human nucleus accumbens. *Cogn Affect Behav Neurosci, 9*(1), 16-27. doi:10.3758/CABN.9.1.16

Braver, T. S. (2012). The variable nature of cognitive control: a dual mechanisms framework. *Trends in Cognitive Sciences, 16*(2), 106-113. doi:https://doi.org/10.1016/j.tics.2011.12.010

Brown, J. W., & Braver, T. S. (2005). Learned Predictions of Error Likelihood in the Anterior Cingulate Cortex. *Science, 307*(5712), 1118-1121. doi:10.1126/science.1105783

Bultena, S., Danielmeier, C., Bekkering, H., & Lemhöfer, K. (2017). Electrophysiological Correlates of Error Monitoring and Feedback Processing in Second Language Learning. *Frontiers in Human Neuroscience, 11*, 29-29. doi:10.3389/fnhum.2017.00029

Frömer, R., Nassar, M. R., Stürmer, B., Sommer, W., & Yeung, N. (2018). I knew that! Confidence in outcome prediction and its impact on feedback processing and learning. *bioRxiv*, 442822. doi:10.1101/442822

Hernandez Lallement, J., Kuss, K., Trautner, P., Weber, B., Falk, A., & Fliessbach, K. (2014). Effort increases sensitivity to reward and loss magnitude in the human brain. *Soc Cogn Affect Neurosci, 9*(3), 342-349. doi:10.1093/scan/nss147

Holroyd, C. B., & Coles, M. G. (2002). The neural basis of human error processing: reinforcement learning, dopamine, and the error-related negativity. *Psychol Rev, 109*(4), 679-709. doi:10.1037/0033-295X.109.4.679

Hughes, G., & Yeung, N. (2011). Dissociable correlates of response conflict and error awareness in error-related brain activity. *Neuropsychologia, 49*(3), 405-415. doi:https://doi.org/10.1016/j.neuropsychologia.2010.11.036

Ma, Q., Meng, L., Wang, L., & Shen, Q. (2014). I endeavor to make it: effort increases valuation of subsequent monetary reward. *Behav Brain Res, 261*, 1-7. doi:10.1016/j.bbr.2013.11.045

Muhlberger, C., Angus, D. J., Jonas, E., Harmon-Jones, C., & Harmon-Jones, E. (2017). Perceived control increases the reward positivity and stimulus preceding negativity. *Psychophysiology, 54*(2), 310-322. doi:10.1111/psyp.12786

Schiffer, A. M., Siletti, K., Waszak, F., & Yeung, N. (2017). Adaptive behaviour and feedback processing integrate experience and instruction in reinforcement learning. *NeuroImage, 146*, 626-641. doi:10.1016/j.neuroimage.2016.08.057

Shenhav, A., Botvinick, M. M., & Cohen, J. D. (2013). The Expected Value of Control: An Integrative Theory of Anterior Cingulate Cortex Function. *Neuron, 79*(2), 217-240. doi:http://dx.doi.org/10.1016/j.neuron.2013.07.007

Shenhav, A., Cohen, J. D., & Botvinick, M. M. (2016). Dorsal anterior cingulate cortex and the value of control. *Nat Neurosci, 19*(10), 1286-1291. doi:10.1038/nn.4384

Wang, L., Zheng, J., & Meng, L. (2017). Effort provides its own reward: endeavors reinforce subjective expectation and evaluation of task performance. *Exp Brain Res, 235*(4), 1107-1118. doi:10.1007/s00221-017-4873-z
