# Supplementary material for: Expectations of reward and efficacy guide cognitive control allocation
Source: Nat Commun. 2021 Feb 15;12:1030. doi: 10.1038/s41467-021-21315-z (PMC7884731; doi:10.1038/s41467-021-21315-z)
Supplement: Supplementary file 3 — Reporting Summary [file 41467_2021_21315_MOESM3_ESM.pdf]

nature research

Corresponding author(s): Romy Froemer
Last updated by author(s): Nov 12, 2020

Reporting Summary

Nature Research wishes to improve the reproducibility of the work that we publish. This form provides structure for consistency and transparency in reporting. For further information on Nature Research policies, see our Editorial Policies and the Editorial Policy Checklist.

Statistics

For all statistical analyses, confirm that the following items are present in the figure legend, table legend, main text, or Methods section.

n/a	Confirmed	
☐	☒	The exact sample size (*n*) for each experimental group/condition, given as a discrete number and unit of measurement
☐	☒	A statement on whether measurements were taken from distinct samples or whether the same sample was measured repeatedly
☐	☒	The statistical test(s) used AND whether they are one- or two-sided<br>*Only common tests should be described solely by name; describe more complex techniques in the Methods section.*
☐	☒	A description of all covariates tested
☐	☒	A description of any assumptions or corrections, such as tests of normality and adjustment for multiple comparisons
☐	☒	A full description of the statistical parameters including central tendency (e.g. means) or other basic estimates (e.g. regression coefficient) AND variation (e.g. standard deviation) or associated estimates of uncertainty (e.g. confidence intervals)
☐	☒	For null hypothesis testing, the test statistic (e.g. *F*, *t*, *r*) with confidence intervals, effect sizes, degrees of freedom and *P* value noted<br>*Give P values as exact values whenever suitable.*
☒	☐	For Bayesian analysis, information on the choice of priors and Markov chain Monte Carlo settings
☒	☐	For hierarchical and complex designs, identification of the appropriate level for tests and full reporting of outcomes
☐	☒	Estimates of effect sizes (e.g. Cohen's *d*, Pearson's *r*), indicating how they were calculated

Our web collection on statistics for biologists contains articles on many of the points above.

Software and code

Policy information about availability of computer code

Data collection	Data were collected using Matlab (R2015b) and the Psychophysics Toolbox (Version 3.0.12), Eyelink algorithm: implemented by the EyeLink 1000 Desktop Mount eye tracker's online parser, which runs on the EyeLink 1000 host software (version 4.56)
Data analysis	Data were analyzed using Matlab and the EEGLab toolbox (Version 14.1.2 and 13.6.5b), EEGLAB iclabel IC classification extension (1Version .1), R (Version 3.6.1) and R-Studio (Version 1.2.1335), lme4 (Version 1.1-21), lmerTest (Version 3.1-1), R.matlab (Version 3.6.2), MASS (Version 7.3-51.5), ggplot2 (Version 3.2.1), psych (1.9.12.31), Hmisc (4.3-1), effects (Version 4.1-4), sjPlot (Version 2.8.2), Rmisc (Version 1.5), Python (Version 3.8.5), Python pypillometry pupil analysis package (1.0.3.1). Custom scripts are available https://osf.io/xuwn9

For manuscripts utilizing custom algorithms or software that are central to the research but not yet described in published literature, software must be made available to editors and reviewers. We strongly encourage code deposition in a community repository (e.g. GitHub). See the Nature Research guidelines for submitting code & software for further information.

Data

Policy information about availability of data

All manuscripts must include a data availability statement. This statement should provide the following information, where applicable:

- Accession codes, unique identifiers, or web links for publicly available datasets
- A list of figures that have associated raw data
- A description of any restrictions on data availability

The datasets generated and analyzed during the current study are available under https://osf.io/xuwn9.

Field-specific reporting

Please select the one below that is the best fit for your research. If you are not sure, read the appropriate sections before making your selection.

- [ ] Life sciences
- [x] Behavioural & social sciences
- [ ] Ecological, evolutionary & environmental sciences

For a reference copy of the document with all sections, see nature.com/documents/nr-reporting-summary-flat.pdf

Behavioural & social sciences study design

All studies must disclose on these points even when the disclosure is negative.

Study description	The studies are quantitative within subject Designs in which efficacy and reward are varied and performance is measured as a function of both variables as well as EEG activity (Study 2).
Research sample	21 individuals participated in Study 1 (age: M = 21.14, SD = 5.15; 17 female), recruited among Brown undergrads and from the general community. 53 University of Toronto undergraduate students (age M = 20.18, SD = 2.30; 15 male; 38 female) participated in study 2. 35 individuals participated in Study 3 (age: M = 20.66, SD = 2.61; 27 female). Study 1 was a pilot study and we subsequently increased the sample size to increase power. The sample size for study 2 was based on a preregistered power analysis. We think that the sample is representative for the question of general mechanisms underlying incentive-driven motivation, while the specific weightings of different incentives are expected to vary meaningfully between different social groups. We thus make claims about the former, but not the latter, for which our samples are not representative.
Sampling strategy	Participants signed up for the experiment. Before data collection for Study 2, we conducted a sensitivity analysis, which indicated that a sample size of N = 50 will provide 80% statistical power to detect effect sizes of d = 0.3 or larger. For studies 1 and 3 sample sizes were not statistically determined, but based on a rule of thumb. While the sample size of study 1 alone would be insufficient, we replicate it in a larger sample in study 2.
Data collection	Participants performed the task on a computer. In Study 2 in addition to the computerized task, EEG-data and pupil data were registered. Nobody besides the researcher and the participant was present during the experiment. The researchers were aware of the within subject manipulations (as indicated by the incentives and known to the participant) and the experimental hypothesis, but not in the room while the participants performed the task.
Timing	Study one: March through April 2017, Study two: March through October 2018, Study 3: April through June 2017
Data exclusions	Across all studies, we excluded participants if they failed performance criteria (i.e., performed below 60% accuracy on high efficacy trials). In Study 1 no participants were excluded. In Study two: We excluded from all analyses 9 participants who performed poorly on the Stroop task , leaving 44 participants in the final sample. The criteria were not mentioned in the preregistration of study two, but had previously been used in the analysis of study 1 data.
Non-participation	no participants dropped out or declined participation
Randomization	All manipulations were within subject, so there were no groups. Conditions were pseudorandomized within subjects.

Reporting for specific materials, systems and methods

We require information from authors about some types of materials, experimental systems and methods used in many studies. Here, indicate whether each material, system or method listed is relevant to your study. If you are not sure if a list item applies to your research, read the appropriate section before selecting a response.

Materials & experimental systems

n/a	Involved in the study	
x		Antibodies
x		Eukaryotic cell lines
x		Palaeontology and archaeology
x		Animals and other organisms
	x	Human research participants
x		Clinical data
x		Dual use research of concern

Methods

n/a	Involved in the study	
x		ChIP-seq
x		Flow cytometry
x		MRI-based neuroimaging

Human research participants

Policy information about studies involving human research participants

Population characteristics	See above
Recruitment	The experiments were advertised using flyers. In Study two, participants who do not want to undergo psychophysiological recordings may have selected to not sign up (negative self-selection). People who are more interested in psychology are more likely o have self-selected to participate in our study.
Ethics oversight	Brown University Institutional Review Board

Note that full information on the approval of the study protocol must also be provided in the manuscript.
